# Effects of different grafting materials on volumetric changes in the Schneiderian membrane following lateral maxillary sinus floor elevation: a preliminary study

**DOI:** 10.1186/s12903-023-02789-3

**Published:** 2023-02-15

**Authors:** Xinke Jiang, Shamin He, Michael M. Bornstein, Yiqun Wu, Lijuan Ye, Feng Wang

**Affiliations:** 1 Department of Oral Implantology, Shanghai Ninth People’s Hospital, Shanghai Jiao Tong University School of Medicine, College of Stomatology, Shanghai Jiao Tong University, National Center for Stomatology, National Clinical Research Center for Oral Diseases, Shanghai Key Laboratory of Stomatology, Shanghai Research Institute of Stomatology, Shanghai, China; 2 Department of Second Dental Center, Shanghai Ninth People’s Hospital, Shanghai Jiao Tong University School of Medicine; College of Stomatology, Shanghai Jiao Tong University; National Center for Stomatology; National Clinical Research Center for Oral Diseases; Shanghai Key Laboratory of Stomatology, Shanghai Research Institute of Stomatology, Shanghai, China; 3 Department of Oral Health & Medicine, University Center for Dental Medicine Basel UZB, University of Basel, Basel, Switzerland

**Keywords:** Schneiderian membrane, Ostium patency, Sinus floor elevation, Volumetric measurement

## Abstract

**Objectives:**

To investigate the potential influence of different grafting materials on maxillary sinus membrane dimensions and ostium patency following lateral sinus floor elevation (SFE) as assessed using cone-beam computed tomography (CBCT).

**Materials and methods:**

A total of 40 sinuses in 40 patients were included. Twenty sinuses were referred for SFE with deproteinized bovine bone mineral (DBBM), and the remaining 20 sinuses were grafted with calcium phosphate (CP). CBCT was performed prior to and 3 to 4 days after surgery. The dimensions of the Schneiderian membrane volume and ostium patency were evaluated, and potential relationships between volumetric changes and any associated factors were analyzed.

**Results:**

The median increase in membrane-whole cavity volume ratios was 43.97% in the DBBM group and 67.58% in the CP group, demonstrating no statistically significant difference (*p* = 0.17). The rates of increased obstruction after SFE were 11.1% for the DBBM group versus 44.4% for the CP group (*p* = 0.03). The graft volume was found to be positively correlated with the postoperative membrane-whole cavity volume ratio (r = 0.79; *p* < 0.01) and the membrane-whole cavity volume ratio increase (r = 0.71; *p* < 0.01).

**Conclusions:**

The two grafting materials seem to have a similar effect on transient volumetric changes in the sinus mucosa. However, the choice of grafting material should still be made with caution since sinuses grafted using DBBM exhibited less swelling and less ostium obstruction.

**Supplementary Information:**

The online version contains supplementary material available at 10.1186/s12903-023-02789-3.

## Background

The maxillary sinus is lined with an epithelial layer, the Schneiderian membrane, which consists of a pseudostratified ciliated respiratory epithelium and acts as a physical barrier and a mechanical clearing system for exogenous agents and particles [1–3]. During implant rehabilitation, bone augmentation is necessary when the vertical bone volume is insufficient at the implant site. It might be necessary to involve the maxillary sinus to increase the bone height for implant insertion in atrophic posterior maxillary areas. The lateral sinus floor elevation (SFE) technique is one of the two main approaches used for sinus floor augmentation [4, 5]. After being gradually modified over the years, the technique is considered a predictable treatment option for the rehabilitation of atrophic posterior maxillae [6–8].

Although laceration and perforation of the membrane should be avoided during the drilling and elevation procedures, the operation carries a potential risk of injuring the sinus membrane. The perforation rate has been reported to be approximately 20%, and several clinical and radiographic variables have been reported to influence the risk of sinus membrane perforation [9, 10]. Griffa et al. evaluated the mucociliary clearance of ten patients undergoing unilateral sinus floor elevation by dropping methylene blue on the sinus floor during the augmentation procedure [11]. Drainage of methylene blue was not observed endoscopically in the detached part of the mucosa immediately after the surgery, showing an absence of mucociliary clearance. Zhou et al. assessed changes in the membrane thickness in 320 sinuses following augmentation [12]. The Schneiderian membrane exhibited significant swelling immediately after the operation, which recovered at 6 months. Patients undergoing endoscopic sinus surgery have shown that mucosal healing and function gradually improved 3 months postoperatively, reflecting the regenerative capability of the sinus membrane [13]. Thus, SFE can be considered traumatic and leads to acute inflammatory reactions of the membrane, with a potential impact on the health of the entire maxillary sinus.

The graft inserted during SFE may also have an impact on membrane healing and sinus physiology. Nevertheless, information on potential reactions of the Schneiderian membrane to different grafting materials is limited. In previous studies [14–17], the physiologic condition of the sinus membrane was assessed by imaging techniques such as cone beam computed tomography (CBCT). Linear measurements were applied as an evaluation parameter, which only assess a limited aspect of the membrane status (e.g. dimensional aspect). It is meaningful to carry out volumetric measurements of the sinus cavity to understand the comprehensive changes in the sinus membrane after different grafting materials have been inserted during SFE. In this study, membrane volume will be proposed as a more dependable parameter to analyze changes and to explore the influence of grafting materials after SFE. This study aimed to compare the volume of the Schneiderian membrane before and 3 to 4 days after lateral SFE using two different bone substitutes and staged implant placement. As a secondary outcome, ostium patency was also assessed following grafting.

## Materials and methods

### Study design and patient selection

All patients included in this study were referred to the Department of Oral Implantology or the Second Dental Center, Ninth People’s Hospital, Shanghai, China, for implant rehabilitation in the atrophic maxillary posterior region from June 2017 to May 2021.

The inclusion criteria for patients were as follows:

- Aged ≥ 18 years
- Systemically healthy and no uncontrolled systemic disease at the time of consultation
- No history of sinus pathology
- Insufficient alveolar bone height at the posterior maxilla (< 4 mm)
- Preoperative CBCT showing a thickness of the sinus membrane <5 mm and no sinus cysts
- No peri-apical lesion of adjacent natural teeth
- Adequate alveolar bone width without horizontal bone augmentation during surgery
- Planned unilateral sinus augmentation using a window and a two-stage approach
- Good maintenance of oral hygiene

The exclusion criteria for patients were as follows:

- Any medical condition that could interfere with the healing process, including pregnancy
- Untreated or uncontrolled periodontitis, periapical diseases or acute sinusitis in/around the surgical field
- Preoperative CBCT showing the thickness of the sinus membrane > 5 mm
- Sinus cyst
- Heavy smokers (>10 cigarettes per day)
- A fully edentulous maxilla
- Preoperative CBCTs were obtained within 3 months before the surgery. Patients were excluded if they had any symptoms such as cold, stuffy nose or nose allergy between the period of preoperative CBCT and surgery.

A clinical controlled nonrandomized study was performed by two experienced surgeons. Every patient provided informed consent before surgery.

From June 2017 until July 2019, twenty consecutive patients fulfilling the inclusion criteria received a deproteinized xenograft (Bio-Oss; Geistlich Pharma AG, Switzerland), comprising the DBBM (deproteinized bovine bone mineral) group. The next 20 consecutive until May 2021 patients fulfilling the inclusion criteria were treated with CP cement (Rebone; Rebone Biomaterials Co., China), thus comprising the CP group.

This study was performed following the principles of the Helsinki Declaration and was approved by the ethics committee of the Ninth People’s Hospital, College of Stomatology, Shanghai Jiao Tong University School of Medicine (NO. SH9H-2021-T187-1).

### Surgical procedures

Surgeries were carried out under adequate local anesthesia. After making a mid-crestal incision and additional vertical releasing incisions, a mucoperiosteal flap was elevated to expose the alveolar crest and the lateral wall of the maxillary sinus. Osteotomy was performed to remove the lateral bony wall using a round bur (Diameter: 2 mm; Institut Straumann AG, Switzerland) outlining the window first, followed by a piezoelectric device. The lateral window was either rectangular or oval in shape. The size of the window was determined based on the extent of the edentulous area and the size of the grafted area and it measured 20 mm × 10 mm on average.

After drilling was completed, the bone window was removed, and the Schneiderian membrane was exposed. The membrane was carefully elevated and dissected from the anterior and medial floors of the sinus cavity with blunt sinus curettes (Dentium advanced sinus kit; Dentium Co. Ltd., Korea). The compartment was then filled with DBBM or CP as the grafting material. No autologous bone was harvested and mixed with the grafting material in all cases. A trimmed collagen membrane (Bio-Gide; Geistlich Pharma AG, Switzerland) was then adapted to cover the bony window. The soft tissues were repositioned and the flaps were closed using 4-0 sutures.

Postoperative oral antibiotics (cefradine 500 mg and metronidazole 400 mg, 3 times daily) and a chlorhexidine oral rinse were prescribed for every patient for 7 days. The sutures were removed 10–14 days after the surgery.

### Imaging procedure and evaluation

Postoperative CBCT scans were obtained 3 to 4 days after the intervention. CBCT images were obtained using the Planmeca Promax Tomography System (Planmeca USA, Inc., USA) with the following operating parameters: 5 mA, 96 kV; voxel size: 0.2 mm; field of view: 13 cm × 9 cm. The primary data were exported as DICOM files and reconstructed in Amira software (Thermo Fisher Scientific Inc., USA) for volumetric measurements of the region of interest (ROI).

**Fig. 1:**
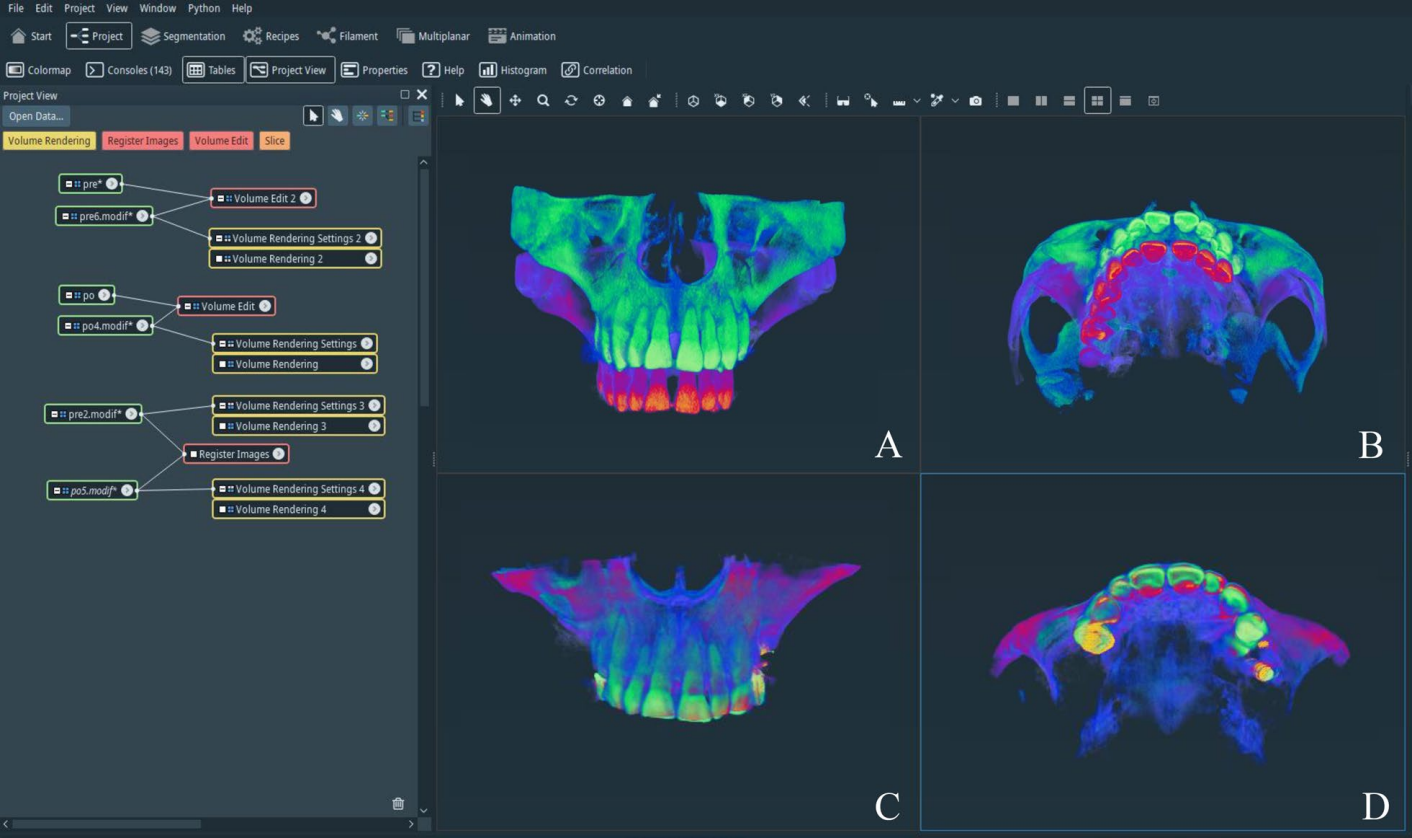
Registration of preoperative and corresponding postoperative CBCT images with 3D reconstruction in the Amira software (Thermo Fisher Scientific Inc., USA). **A** Coronal view of the preoperative (Red) and postoperative (Green) CBCT images before registration; **B** Axial view of the preoperative and postoperative CBCT images before registration; **C** Coronal view of the registered maxilla; **D** Axial view of the registered maxilla

**Fig. 2:**
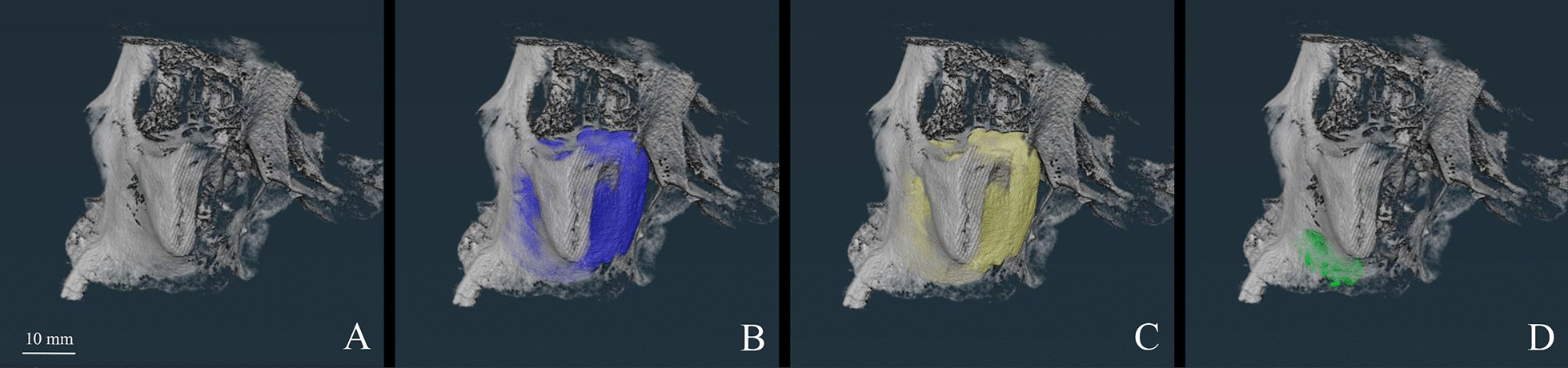
Three-dimensional display of the measurement. **A** Three-dimensional reconstruction of CBCT data; **B** The whole sinus cavity (purple); **C** The air cavity (yellow) of the preoperative sinus; **D** The grafts (Green) in the sinus cavity extracted from post-operative CBCT

**Fig. 3:**
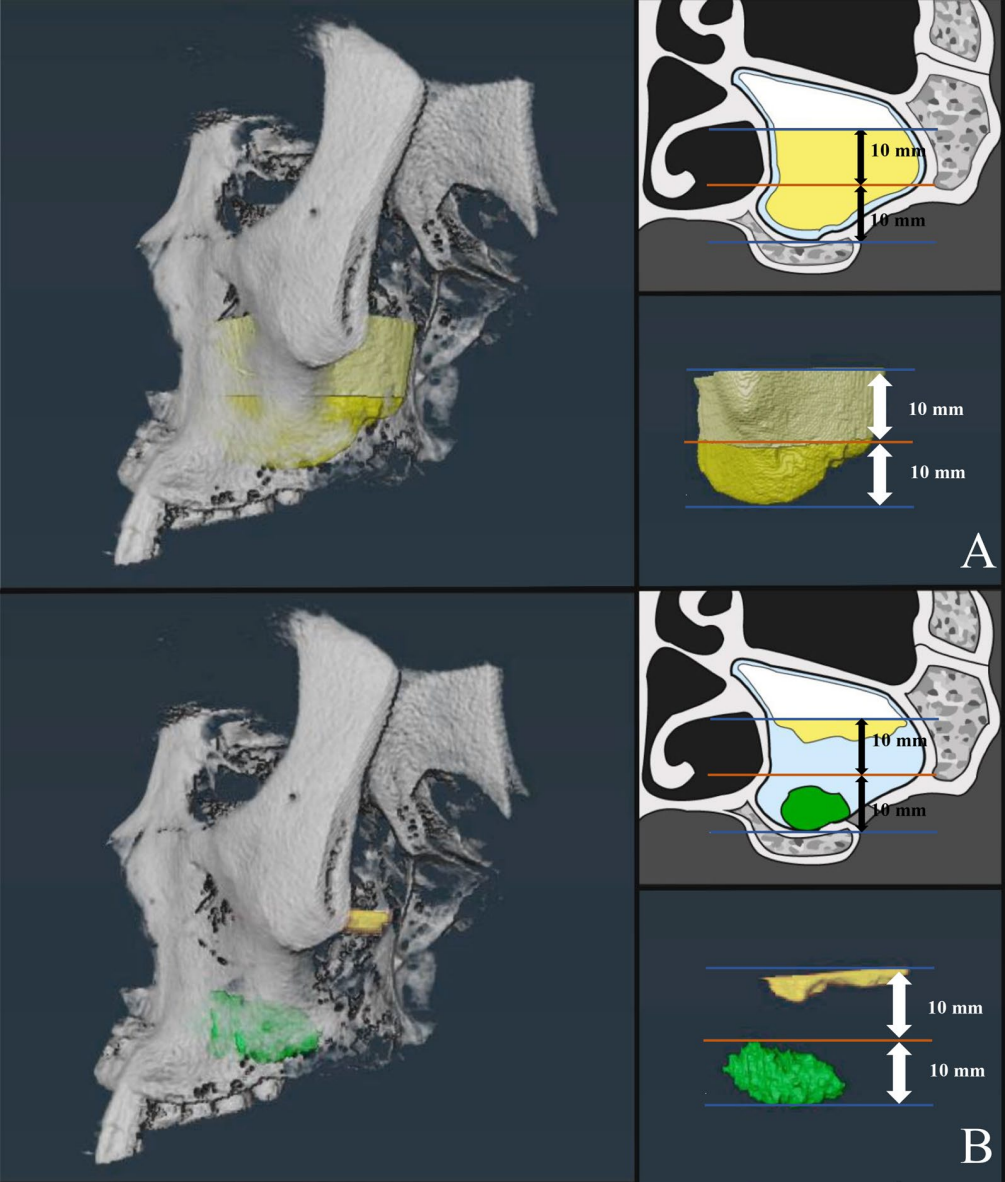
Preoperative and corresponding postoperative segmentation with 3D reconstruction. The bottom blue line indicates the bottom of sinus, and the orange line indicates the height of grafts. The upper blue line is the upper limit of the measurement range, located 20 mm above the bottom blue line. **A** The yellow part indicates the air cavity of the preoperative sinus and the blue part indicates the preoperative sinus membrane. **B** The yellow part indicates the air cavity of the postoperative sinus, the green part indicates the graft, and the blue part indicates the swelling membrane

Three steps were taken to ensure measurement consistency. The Volume Edit function was utilized to crop out the mandible and cervical vertebra to avoid interference with the image registration. The function of Register Images with the Normalized Mutual Information mode was then utilized to match the nonoperated bony regions of the preoperative CBCT and corresponding postoperative CBCT images (Fig. 1). Third, the Resample Transformed Image function was applied to reorient the axial slices of both CBCT scans to be parallel to the plane of the palatal bone. With the software the volume including the whole sinus cavity, air cavity and grafts could be reconstructed and displayed (Fig. 2). Considering the limited scanning area of the operated sinus and the height of the grafts, the axial slice (slice x), which was tangential to the deepest point of the inner surface of the sinus, was set as the bottom of the ROI, and the upper limit of the measurement range was set at a 20 mm level above the bottom line, twice the height of the graft (slice (x + 100)). In the coronal slices, the grafting material, the air cavity and the whole cavity of the sinus were selected as structures of interest using the semiautomated segmentation tool (Fig. 3). These parts were extracted through the watershed algorithm function and reconstructed and measured through the Arithmetic, Label Analysis and Volume Rendering functions.

The following parameters of the pre- and postoperative maxillary sinuses were assessed:

1. The volume of the grafted material: VGM
2. The volume of the air cavity: VACpre and VACpost
3. The preoperative volume of whole cavity volume: VWCpre

The postoperative volume of the whole cavity (VWCpost) was calculated as follows:

$${\text{VWCpost }} = {\text{ VWCpre }} - {\text{ VGM}}$$

The pre- and postoperative volumes of the Schneiderian membrane (VSM) were calculated as follows:

$${\text{VSMpre }} = {\text{ VWCpre }}{-}{\text{ VACpre}}$$

$${\text{VSMpost }} = {\text{VWCpost }}{-}{\text{ VACpost}}$$

The pre- and postoperative membrane-whole cavity volume ratios were calculated as follows:

$${\text{Rpre }} = {\text{ VSMpre}}/{\text{VWCpre}}$$

$${\text{Rpost }} = {\text{ VSMpost}}/{\text{VWCpost}}$$

The difference between the postoperative R and the preoperative R was calculated as follows:

$${\text{D }} = {\text{ Rpost }}{-}{\text{ Rpre}}$$

Relevant measurements were completed by two independent examiners who were blinded to the study groups. Inter-examiner reliability was ensured by measuring the membrane volume of 5 sinuses separately to achieve an intraclass correlation coefficient (ICC) of 0.90. An ICC of 0.96 was obtained using a two-way random-effects model with an absolute agreement definition, showing very reliable agreement. The 2 included groups of cases (40 in total) were separately assessed by the two observers. If a difference greater than 100 mm^3^ occurred, a third examiner was consulted, and additional measurements were performed to reach a final consensus.

The ostium condition of the maxillary sinus was also set as an evaluation parameter. The patency of the ostium was evaluated by two observers for preoperative and postoperative CBCT slices using coronal plane. Patency was classified as "patent" or "obstructed".

### Statistical analysis

The obtained data were organized and loaded into SPSS 22.0 software (IBM, New York, USA). The sinus was regarded as a separate unit when the analysis was performed. For a descriptive analysis of the continuous data, the normality of the data was examined by the Shapiro–Wilk test, and the homogeneity of variance was examined by Levene's test. For continuous data not following a normal distribution, the median and interquartile range (IQR) were used in addition to the mean and standard deviation.

To analyze volumetric changes in the mucosa, D values (the changes in the membrane-whole cavity volume ratio) were extracted to examine whether they were significantly different in the two groups by the Wilcoxon signed-rank test. The chi-squared test was used to examine significant differences in the occurrence of ostium obstructions. Correlation analysis was performed to study the association between sex, age, the number of missing teeth in the grafted region, the graft volume and the VSMpost, Rpost, D, and the patency of the ostium after surgery. A significance level of 5% (*p <* 0.05) was accepted in all statistical tests.

## Results

### Clinical report

From June 2017 to May 2021, a total of 40 patients met the inclusion criteria; 20 patients with 20 sinuses received DBBM, while the other half received CP cement during sinus floor elevation. Preoperative and 3-4-day postoperative CBCT data were obtained from these patients. The detailed clinical characteristics of the included patients are shown in Table 1.

**Table 1:** Clinical characteristics of the patients in the deproteinized bovine bone mineral (DBBM) group and the calcium phosphate (CP) group.

	DBBM (N=20)	CP (N=20)
Gender (male/female)	11/9	9/11
Age (Mean ± SD; Range)	47.2 ± 13.9; 29–79	46.8 ± 12.1; 19–66
No. of missing teeth in grafting region (Mean ± SD; Range)	2.3 ± 1.2; 1–4	2.2 ± 1.0; 1–4
Quantity (g) of grafting material (Mean ± SD; Range)	1.33 ± 0.64; 0.5–2.5	1.23 ± 0.53; 0.5–2.5

There was no sinus membrane perforation or laceration during surgery for any of the included cases. Wound healing was satisfactory in all patients and exhibited no relevant symptoms of postoperative infections, including local pain and tenderness, nasal obstruction, flap dehiscence, or suppuration.

### Volumetric measurements and correlation analysis

The median preoperative volumes of the Schneiderian membrane (VSMpre) in the DBBM and CP groups were 910.52 mm^3^ and 796.14 mm^3^, respectively. This value increased to 5341.71 mm^3^ in the DBBM group and 5269.44 mm^3^ in the CP group 3 to 4 days after SFE (Additional file 1: Table S1). A statistically significant increase in membrane volume was observed in the DBBM group relative to the CP group (*p* < 0.05).

In the postoperative CBCT measurements, the median graft volume was 1243.98 mm^3^ for DBBM (IQR: (1018.66, 1949.15)), and 1564.17 mm^3^ for CP (IQR: (1053.40, 1756.76)) (Additional file 1: Table S1). No significant difference was found for the grafting volume between the two groups (*p* = 0.78).

**Fig. 4:**
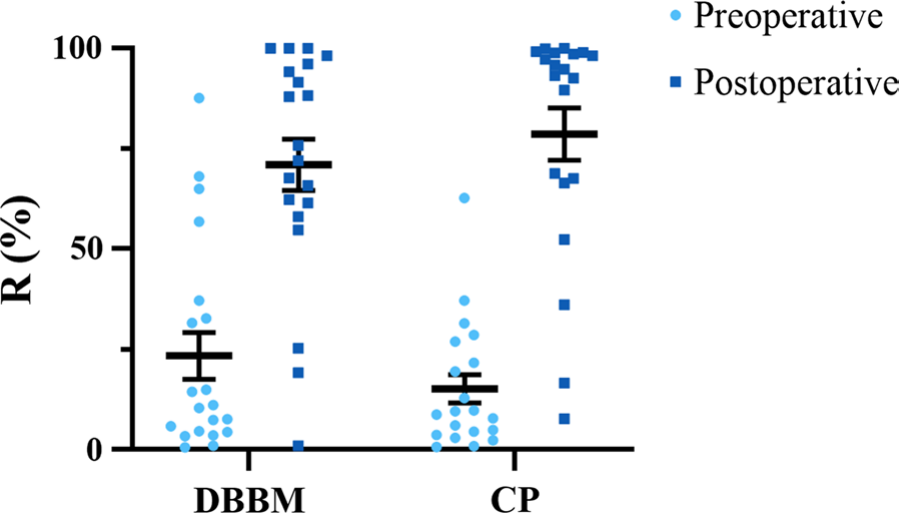
Distribution of preoperative and postoperative membrane-bone cavity volume ratio (R) in the two groups. DBBM: deproteinized bovine bone material; CP: calcium phosphate

R represents the membrane-whole cavity volume ratio. The pre- and post-R distribution of the two groups was shown in Figure 4. The percentage of swollen sinuses (R > 90%) rose from 0 to 35% and 0 to 60% after surgery in the two groups (Additional file 1: Table S2), respectively, a difference that was significant (*p* = 0.001).

The value of D (the change between the post- and preoperative membrane-whole cavity volume ratio) ranged from 0.43% to 96.67% in the DBBM group and from 1.71% to 99.10% in the CP group (Table 2). There was no significant difference in the D value between the two groups (*p* = 0.17).

**Table 2:** Difference between the postoperative and preoperative membrane-whole cavity volume ratio (D) in the two groups.

D (%)	DBBM	CP
^*^Median	43.97	67.58
P25	21.17	52.49
P75	82.55	86.63
Mean	47.56	63.47
SD	34.44	27.01

^*^p = 0.17

DBBM: deproteinized bovine bone material; CP: calcium phosphate.

Correlation analysis was performed to determine the potential effect of relevant variables on the volumetric change of the sinus membrane. Rpost (*r* = 0.789; *p* <.001) and D (*r* = 0.705; *p* <.001) were found to have a positive correlation with graft volume, signifying that a larger volume of grafting material was correlated with more severe swelling of the sinus membrane. No significant correlation was noted between Rpost and the gender, age, or the number of missing teeth in the grafted region (*p* = 0.218, 0.358, 0.078, respectively) or between D and the gender, age or the number of missing teeth in the grafted region (*p* = 0.533, 0.387, 0.106, respectively).

### Ostium patency

**Fig. 5:**
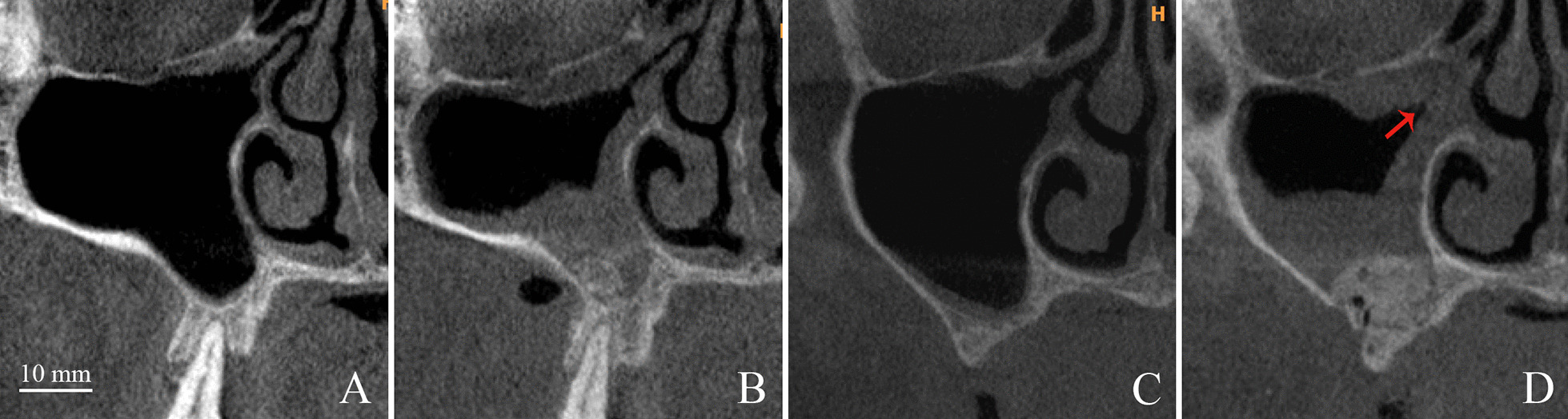
The coronal view of preoperative and postoperative maxillary sinuses grafted with deproteinized bovine bone material (**A** and **B**) and calcium phosphate (**C** and **D**). The membranes in the maxillary sinus floor and the infundibulum swell 3 days after lateral sinus floor elevation (**B** and **D**). The red arrow in (**D**) indicates ostial obstruction

Additionally, based on the preoperative CBCT assessment, the patency of the ostia in 2 cases in each group was not clearly visible on all three axes, although the overall thickness of the sinus membrane was <5 mm in all enrolled cases. Therefore, 18 cases per group were used for analyzing the status of the ostia postoperatively (Fig. 5). Two and 8 obstructed ostia were detected 3 to 4 days after augmentation in the DBBM and CP groups, respectively. Pearson’s chi-square test revealed that the incidence of ostium obstruction in the DBBM group was significantly lower than that in the CP group (*χ2* = 4.985*, p* = 0.026).

## Discussion

The present study revealed no difference in the transit postoperative volumetric changes of the sinus membrane between the two different grafting materials, indicating that the sinus mucosa might have a similar physiological response to the stimulus from both materials. However, it should be noted that sinuses grafted with DBBM exhibited less swelling and fewer ostium obstructions overall.

Radiographic transit sinus membrane swelling after SFE has been observed in several studies [12, 15, 16, 18]. It was believed that the thickening might be caused by a physiologic inflammatory reaction due to surgical trauma involving bleeding and swelling during the initial healing phases. Temmerman et al. assessed the volume of the membrane after lateral SFE, transcrestal (osteotomy) SFE and intrasinus (piezosurgery) SFE procedures and compared the volumetric changes directly [19]. No statistically significant differences could be observed among these three treatment options with regard to the swelling of the Schneiderian membrane 6 days after surgery. In that study, deproteinized bovine bone mineral (DBBM) mixed with PRF (platelet-rich fibrin) was added to the antrum. Thus, it seems that different surgical approaches to the sinus while using the same grafting material resulted in a similar membrane response. When a resorbable bone substitute was used in a goat model, major histologic modifications of the sinus membrane were observed, including an increase in goblet cells and a decrease in the number of glands with mucous and serous cells and ciliated cells [20]. Thus, it seems worthwhile to study the effects of the different grafting materials used for SFE on the status of the Schneiderian membrane and the entire maxillary sinus.

The thickness of the membrane varies in various regions of the maxillary sinus. Several studies adopted linear (2-dimensional) measurements at different points of interest [21–23]. However, linear measurements only indicate a regional and limited status of the membrane. To understand the comprehensive changes in the sinus membrane after SFE, it is meaningful to carry out volumetric (3-dimensional) measurements of the sinus cavity.

In the present study, volumetric measurements were carried out using dedicated software. The height of the graft was approximately 10 mm, which was not significantly different from the results in previous studies [15, 21]. To focus on the physiological response of the membrane around the grafting material, the upper limit of the measurement range was set at a 20 mm level above the sinus floor, twice the height of the graft. The difference in individual maxillary sinus volume leads to a difference in mucosal volume. Therefore, to eliminate the interference of the size of the sinus, in the present study, the ratio of the membrane to sinus volume was chosen to assess the changes in the two groups.

Several time points were set to measure the changes of the membrane thickness over time in previous studies. Guo et al. and Zhou et al. reported that membranes swell immediately after SFE [12, 14]. The significant change of membrane thickness was also observed at 7 days after SFE [16, 18, 19]. However, the true peak of membrane swelling after surgery is not well understood.

Makary et al. assessed 31 maxillary sinuses in 24 patients and observed that preoperative mean membrane thickness was 0.75 mm [15]. It significantly increased in groups assessed respectively at day 1 (2.36 mm), 2 (4.14 mm), 3 (6.05 mm), and 7 (6.63 mm) following SFE. The results should be interpreted with caution due to the limited sample size and the heterogeneity among groups. In consideration of ethical limitations on radiation exposure, it is impossible to track dynamic sinus membrane changes over a short period time after SFE in the same patient using ionizing imaging methods. In the present study, a time point for observation and measurement according to the previously reported ‘initial peak’ period was selected to understand the short-term influence of different grafting materials on the maxillary sinus membrane dimensions and ostium patency.

Although the analysis showed no significant difference between the differences in volume ratio, indicating that the impact of the two materials on the mucosa may not be significantly different, it cannot be ignored that the volume ratio was within 90-100% postoperatively in 12 of 20 patients in the group with CP, while in the group with DBBM, it was 7 of 20. A value of 90%-100% indicates a severe condition of membrane swelling. This usually means that the membrane was at least 2 cm above the bone level initially measured, which should also be considered when surgeons choose an appropriate grafting material for SFE.

A significant positive correlation was observed between the graft volume and the proportion of transient membrane volume in the whole cavity in the present study. This observation indicates that a larger sinus floor elevation with more grafting material may lead to a more significant response of the sinus membrane shortly after surgery. Thus, there is clearly a higher risk of blocking the ostium, especially when an increased thickness of the Schneiderian membrane was already present preoperatively [24].

Mucociliary clearance is a primary defense mechanism of the respiratory tract, and the health of the maxillary sinus is primarily dependent on effective mucociliary clearance [25]. Park et al. reported that mucociliary clearance allowed the ventilation of displaced graft particles through a perforated Schneiderian membrane, and did not observe particles in the maxillary sinus 1 week after maxillary sinus augmentation on CBCT scans [26]. In the present study, no perforation-related adverse event was observed, and no acute maxillary sinusitis with impaired mucociliary function was reported.

The infundibulum connecting the maxillary sinus and the nasal cavity plays an important role in the physiological condition of the maxillary sinus [27]. When ostium obstruction occurs, the draining and mucosal clearance of the sinus may be compromised, which might cause pathologic changes such as acute or chronic sinusitis. According to the data included in the present study, postoperative swelling, which increases the mucosa volume, might reach the region of the ostium and cause obstruction. Guo et al. reported an increase of 14% in the ostium obstruction rate (from 16% to 30%) in 53 cases immediately after sinus augmentation with a DBBM graft [14], and Sakuma et al. reported an increase rate of 15% (from 4% to 19%) one week after surgery with two different xenografts [18]. Park et al. evaluated patency/obstruction of the ostium in patients with mucosal thickening, and did not find a statistically significant influence of ostial patency before lateral SFE surgery on the membrane thickness change after prosthesis delivery [17]. In the present study, the DBBM group appeared to have a significantly lower incidence of ostium obstruction than the CP group after augmentation. However, from clinical point of view, all patients in both groups did not exhibit nasal obstruction and relevant symptoms of postoperative infections. Therefore, the histological response of the membrane to different grafting materials should be further studied. On the other hand, it is noteworthy that the relationship between the duration of ostium obstruction and the incidence of sinus infection after surgery.

In the maxillary sinus as well as other anterior sinuses, the mucociliary pathway commonly leads to the ostiomeatal complex, which plays a significant role in the ventilation and drainage of sinuses [25]. Anatomical variations of the osteomeatal complex, including stenosis of ostium, could have deleterious effects on the sinus physiology as the permeability of ostium is altered [28]. In previous studies, ostium obstruction was found to be not only associated with inflammatory or neoplastic factors, but also correlated with a congenital stenosis [29]. In the present study, the qualified CBCT scans of 36 participants did not show a preoperative stenosis of the osteomeatal complex, which must be further evaluated with a larger sample size.

Although CBCT data have been utilized widely in studies evaluating the maxillary sinus membrane, some limitations cannot be ignored. The most important include the inability to distinguish liquids from soft tissue, which may lead to larger volume measurements. The size of the spatial resolution is also a potential interfering factor, and the 0.2 mm voxel size of our included data allows an inherent measurement error of ± 0.2 mm. Thus, the results in the present study should be interpreted with some caution since the error may be amplified when using three-dimensional measurements. Although the volumetric change revealed no statistically significant difference between the two groups, a strong conclusion cannot be drawn despite the sinuses being less swollen and exhibiting less ostium obstruction in the DBBM group. In the future, longitudinal studies with long-term follow-up are needed to explore whether different materials have different influences on the recovery rate of mucosal swelling and ostium obstruction.

## Conclusion

Within the limits of the preliminary study, DBBM and CP seem to have similar effects on the postoperative volumetric changes of the Schneiderian membrane. However, the choice of grafting material may still need to be made with caution since sinuses grafted using DBBM exhibited less swelling and there was less ostium obstruction. Future studies with a larger sample size and a longer follow-up are needed to draw more definitive conclusions.

## Supplementary Information

**Additional file 1.** **Table S1.** The results of volumetric measurement in the two groups. **Table S2.** Distribution of preoperative and postoperative membrane-bone cavity volume ratio (R) in the two groups.

## Data Availability

The data and materials collected in this research are available from the corresponding author when requested reasonably.

## Abbreviations

SFE: Sinus floor elevation
CBCT: Cone-beam computed tomography
DBBM: Deproteinized bovine bone mineral
CP: Calcium phosphate
ICC: Intraclass correlation coefficient
IQR: Interquartile range

## References

[1] Torkkeli T, Rautiainen M, Nuutinen J (1994). Ciliary ultrastructure and mucociliary transport in upper respiratory tract infections. Am J Rhinol.

[2] Pommer B, Unger E, Sütö D, Hack N, Watzek G (2009). Mechanical properties of the Schneiderian membrane in vitro. Clin Oral Implants Res.

[3] Testori T, Weinstein T, Taschieri S, Wallace SS: Risk factors in lateral window sinus elevation surgery. Periodontol 2000 2019, 81(1). 10.1111/prd.12286 31407430

[4] Boyne PJ, James RA (1980). Grafting of the maxillary sinus floor with autogenous marrow and bone. J Oral Surg.

[5] Tatum H (1986). Maxillary and sinus implant reconstructions. Dent Clin North Am.

[6] Lundgren S, Cricchio G, Hallman M, Jungner M, Rasmusson L, Sennerby L (2017). Sinus floor elevation procedures to enable implant placement and integration: techniques, biological aspects and clinical outcomes. Periodontol 2000.

[7] Park WB, Kang KL, Han JY (2019). Factors influencing long-term survival rates of implants placed simultaneously with lateral maxillary sinus floor augmentation: A 6- to 20-year retrospective study. Clin Oral Implants Res.

[8] Ha J, Son JH, Sung IY, Cho YC, Choi JH (2020). Clinical outcome of implants placed in grafted maxillary sinus via lateral approach: a 10-year follow-up study. J Dent Sci.

[9] Krennmair S, Malek M, Forstner T, Krennmair G, Weinländer M, Hunger S (2020). Risk factor analysis affecting sinus membrane perforation during lateral window maxillary sinus elevation surgery. Int J Oral Maxillofac Implants.

[10] Shao Q, Li J, Pu R, Feng Y, Jiang Z, Yang G (2021). Risk factors for sinus membrane perforation during lateral window maxillary sinus floor elevation surgery: a retrospective study. Clin Implant Dent Relat Res.

[11] Griffa A, Berrone M, Boffano P, Viterbo S, Berrone S (2010). Mucociliary function during maxillary sinus floor elevation. J Craniofac Surg.

[12] Zhou C, Sun SZ, Lin MN, He FM (2021). Responses of sinus membrane and antral pseudocyst following lateral window sinus augmentation with bone grafting: a retrospective study. Int J Oral Maxillofac Implants.

[13] Inanli S, Tutkun A, Batman C, Okar I, Uneri C, Sehitoğlu MA (2000). The effect of endoscopic sinus surgery on mucociliary activity and healing of maxillary sinus mucosa. Rhinology.

[14] Guo Z-Z, Liu Y, Qin L, Song Y-L, Xie C, Li D-H (2016). Longitudinal response of membrane thickness and ostium patency following sinus floor elevation: a prospective cohort study. Clin Oral Implants Res.

[15] Makary C, Menhall A, Rebaudi A (2019). Early postoperative reactions following lateral sinus floor elevation using piezosurgery: a radiographic study. Clin Implant Dent Relat Res.

[16] Kato S, Omori Y, Kanayama M, Hirota A, Ferri M, Apaza Alccayhuaman KA, Botticelli D: Sinus mucosa thickness changes and ostium involvement after maxillary sinus floor elevation in sinus with septa. A cone beam computed tomography study. Dentistry J 2021, 9(8). 10.3390/dj9080082 PMC8391700 34435994

[17] Park WB, Kim J, Kim YJ, Kang P, Lim HC, Han JY: Changes in sinus mucosal thickening in the course of tooth extraction and lateral sinus augmentation with surgical drainage: A cone-beam computed tomographic study. Clin Oral Implants Res 2022. 10.1111/clr.14019 36336985

[18] Sakuma S, Ferri M, Imai H, Fortich Mesa N, Blanco Victorio DJ, Apaza Alccayhuaman KA, Botticelli D (2020). Involvement of the maxillary sinus ostium (MSO) in the edematous processes after sinus floor augmentation: a cone-beam computed tomographic study. Int J Implant Dent.

[19] Temmerman A, Van Dessel J, Cortellini S, Jacobs R, Teughels W, Quirynen M (2017). Volumetric changes of grafted volumes and the Schneiderian membrane after transcrestal and lateral sinus floor elevation procedures: a clinical, pilot study. J Clin Periodontol.

[20] Bravetti P, Membre H, Marchal L, Jankowski R (1998). Histologic changes in the sinus membrane after maxillary sinus augmentation in goats. J Oral Maxillofac Surg.

[21] Anduze-Acher G, Brochery B, Felizardo R, Valentini P, Katsahian S, Bouchard P (2013). Change in sinus membrane dimension following sinus floor elevation: a retrospective cohort study. Clin Oral Implants Res.

[22] García-Denche JT, Wu X, Martinez P-P, Eimar H (2013). Ikbal DJ-A, Hernández G, López-Cabarcos E, Fernandez-Tresguerres I, Tamimi F: Membranes over the lateral window in sinus augmentation procedures: a two-arm and split-mouth randomized clinical trials. J Clin Periodontol.

[23] Shanbhag S, Karnik P, Shirke P, Shanbhag V (2014). Cone-beam computed tomographic analysis of sinus membrane thickness, ostium patency, and residual ridge heights in the posterior maxilla: implications for sinus floor elevation. Clin Oral Implants Res.

[24] Janner SFM, Caversaccio MD, Dubach P, Sendi P, Buser D, Bornstein MM (2011). Characteristics and dimensions of the Schneiderian membrane: a radiographic analysis using cone beam computed tomography in patients referred for dental implant surgery in the posterior maxilla. Clin Oral Implants Res.

[25] Whyte A, Boeddinghaus R (2019). The maxillary sinus: physiology, development and imaging anatomy. Dentomaxillofac Radiol.

[26] Park WB, Cho NJ, Kang P: Tomographic imaging of mucociliary clearance following maxillary sinus augmentation: a case series. Medicina (Kaunas, Lithuania) 2022, 58(5). 10.3390/medicina58050672 PMC9145337 35630089

[27] Timmenga NM, Raghoebar GM, Liem RSB, van Weissenbruch R, Manson WL, Vissink A (2003). Effects of maxillary sinus floor elevation surgery on maxillary sinus physiology. Eur J Oral Sci.

[28] Almaghrabi BA, Hatton MN, Andreana S, Hoeplinger MA (2011). Treatment of severe sinus infection after sinus lift procedure: a case report. Implant Dent.

[29] Beaumont C, Zafiropoulos GG, Rohmann K, Tatakis DN (2005). Prevalence of maxillary sinus disease and abnormalities in patients scheduled for sinus lift procedures. J Periodontol.

