# Supplementary material for: Effects of different grafting materials on volumetric changes in the Schneiderian membrane following lateral maxillary sinus floor elevation: a preliminary study
Source: BMC Oral Health. 2023 Feb 15;23:102. doi: 10.1186/s12903-023-02789-3 (PMC9933314; doi:10.1186/s12903-023-02789-3)
Supplement: Supplementary file 1 — Additional file 1. Table S1. The results of volumetric measurement in the two groups. Table S2. Distribution of preoperative and postoperative membrane-bone cavity volume ratio (R) in the two groups. [file 12903_2023_2789_MOESM1_ESM.docx]

**Table S1**

The results of volumetric measurement in the two groups.

| Patient No. | Group | VSMpre (mm^3^) | VSMpost (mm^3^) | VGM (mm^3^) | VWCpre (mm^3^) |
| --- | --- | --- | --- | --- | --- |
| 1 | DBBM | 1175.80 | 7456.89 | 2627.50 | 10546.37 |
| 2 | DBBM | 70.17 | 5271.48 | 1448.21 | 7440.91 |
| 3 | DBBM | 4310.53 | 6559.36 | 900.80 | 11583.93 |
| 4 | DBBM | 1147.83 | 1286.36 | 1275.86 | 7971.34 |
| 5 | DBBM | 467.98 | 6110.56 | 1739.88 | 10228.13 |
| 6 | DBBM | 673.20 | 6781.25 | 2108.99 | 8890.24 |
| 7 | DBBM | 2505.87 | 3769.83 | 749.67 | 7643.38 |
| 8 | DBBM | 594.39 | 2873.97 | 1077.40 | 5690.22 |
| 9 | DBBM | 3084.67 | 5411.94 | 1764.93 | 9762.27 |
| 10 | DBBM | 274.63 | 6233.25 | 2010.56 | 8243.81 |
| 11 | DBBM | 4547.17 | 3900.86 | 770.31 | 5188.48 |
| 12 | DBBM | 513.93 | 4454.90 | 2399.16 | 6938.29 |
| 13 | DBBM | 3628.97 | 4520.07 | 1063.51 | 5583.58 |
| 14 | DBBM | 354.25 | 5735.28 | 1212.09 | 9930.12 |
| 15 | DBBM | 509.18 | 5770.83 | 1065.26 | 8678.48 |
| 16 | DBBM | 349.75 | 1361.41 | 2570.41 | 7945.63 |
| 17 | DBBM | 7157.48 | 8316.31 | 1437.28 | 10523.22 |
| 18 | DBBM | 50.93 | 79.72 | 802.78 | 8672.30 |
| 19 | DBBM | 1543.82 | 8808.86 | 1126.23 | 10288.94 |
| 20 | DBBM | 3975.29 | 3479.20 | 1003.71 | 7000.25 |
| 21 | CP | 10811.76 | 15552.22 | 1682.55 | 17240.57 |
| 22 | CP | 153.74 | 2598.26 | 1722.60 | 6691.34 |
| 23 | CP | 2110.29 | 8985.56 | 1763.22 | 10821.81 |
| 24 | CP | 60.13 | 6878.25 | 1063.76 | 8737.79 |
| 25 | CP | 252.82 | 4689.99 | 697.14 | 5592.69 |
| 26 | CP | 418.85 | 448.62 | 1154.94 | 6952.48 |
| 27 | CP | 909.27 | 5578.81 | 1015.53 | 7042.81 |
| 28 | CP | 746.05 | 938.09 | 2011.85 | 7666.39 |
| 29 | CP | 921.82 | 2980.22 | 3548.82 | 11789.20 |
| 30 | CP | 652.87 | 5268.15 | 1472.01 | 6791.37 |
| 31 | CP | 3037.61 | 6311.86 | 1664.84 | 8156.17 |
| 32 | CP | 466.38 | 5639.61 | 1049.95 | 9402.58 |
| 33 | CP | 1459.95 | 4719.40 | 1656.32 | 6716.95 |
| 34 | CP | 249.22 | 4039.83 | 867.49 | 6739.77 |
| 35 | CP | 846.22 | 7803.30 | 1737.38 | 9649.44 |
| 36 | CP | 190.41 | 3943.30 | 486.38 | 6428.55 |
| 37 | CP | 2369.94 | 5194.47 | 2267.36 | 7519.07 |
| 38 | CP | 104.67 | 9655.69 | 1959.94 | 11615.63 |
| 39 | CP | 1837.99 | 5270.73 | 1249.19 | 6812.11 |
| 40 | CP | 2237.36 | 6244.92 | 1465.53 | 7828.33 |

DBBM: deproteinized bovine bone material; CP: calcium phosphate; VMCpre and VMCpost: the pre and postoperative volume of the Schneiderian membrane; VGM: the volume of the grafted material; VWCpre: the preoperative volume of whole cavity volume.

**Table S2**

Distribution of preoperative and postoperative membrane-bone cavity volume ratio (R) in the two groups.

|  | DBBM | | | | CP | | | |
| --- | --- | --- | --- | --- | --- | --- | --- | --- |
|  | Preoperative | | Postoperative | | Preoperative | | Postoperative | |
|  |  |  |  |  |  |  |  |  |
| R  (%) | frequency | Proportion (%) | frequency | Proportion (%) | frequency | Proportion (%) | frequency | Proportion (%) |
| 0-20 | 14 | 70 | 2 | 10 | 14 | 70 | 2 | 10 |
| 20-80 | 6 | 30 | 9 | 45 | 6 | 30 | 5 | 25 |
| 80-90 | 0 | 0 | 2 | 10 | 0 | 0 | 1 | 5 |
| 90-100 | 0 | 0 | 7 | 35 | 0 | 0 | 12 | 60 |

DBBM: deproteinized bovine bone material; CP: calcium phosphate.
